# Does poverty alleviation decrease depression symptoms in post-conflict settings? A cluster-randomized trial of microenterprise assistance in Northern Uganda

**DOI:** 10.1017/gmh.2015.28

**Published:** 2016-02-29

**Authors:** E. P. Green, C. Blattman, J. Jamison, J. Annan

**Affiliations:** 1 Duke Global Health Institute, Box 90519, Durham, North Carolina 27708, USA; 2 Columbia University, School of International & Public Affairs (SIPA); 3 Global Insights Initiative at the World Bank; 4 International Rescue Committee

**Keywords:** Poverty, cash transfers, Africa, field experiment, depression

## Abstract

**Background.:**

By 2009, two decades of war and widespread displacement left the majority of the population of Northern Uganda impoverished.

**Methods.:**

This study used a cluster-randomized design to test the hypothesis that a poverty alleviation program would improve economic security and reduce symptoms of depression in a sample of mostly young women. Roughly 120 villages in Northern Uganda were invited to participate. Community committees were asked to identify the most vulnerable women (and some men) to participate. The implementing agency screened all proposed participants, and a total of 1800 were enrolled. Following a baseline survey, villages were randomized to a treatment or wait-list control group. Participants in treatment villages received training, start-up capital, and follow-up support. Participants, implementers, and data collectors were not blinded to treatment status.

**Results.:**

Villages were randomized to the treatment group (60 villages with 896 participants) or the wait-list control group (60 villages with 904 participants) with an allocation ration of 1:1. All clusters participated in the intervention and were included in the analysis. The intent-to-treat analysis included 860 treatment participants and 866 control participants (4.1% attrition). Sixteen months after the program, monthly cash earnings doubled from UGX 22 523 to 51 124, non-household and non-farm businesses doubled, and cash savings roughly quadrupled. There was no measurable effect on a locally derived measure of symptoms of depression.

**Conclusions.:**

Despite finding large increases in business, income, and savings among the treatment group, we do not find support for an indirect effect of poverty alleviation on symptoms of depression.

Poverty and mental ill health are often comorbid. One in five people in developing countries lives in extreme poverty (UN, 2014), and mental and behavioral disorders account for nearly a quarter of the disability burden in these countries (GBD, 2010, 2012). The majority of the world's poor, however, do not suffer from mental illness. Thus, the relationship between poverty and mental ill health is complex. How we understand this association has important implications for policy.

Several reviews have evaluated whether or not the evidence base supports a link between poverty and mental ill health. Patel & Kleinman (2003) reviewed selected epidemiological studies in low- and middle-income countries and argued that evidence demonstrates a clear association between poverty and common mental disorders (CMD). In contrast, Das *et al*. (2007) examined the results of several nationally representative surveys conducted in similar settings and concluded that the link between poverty and poor mental health is weak and inconsistent. In particular, the authors reported that they found no association between consumption poverty and mental health. In the most comprehensive review on the topic surveying 115 studies conducted in low- and middle-income countries, Lund *et al*. (2010) also found that the direction and strength of the poverty–mental health relationship varies across studies, but concluded that the totality of evidence suggested that some dimensions of poverty – for instance, education, food insecurity, financial stress, social class – are consistently related to CMD; however, the association between CMD and other measures of poverty, such as income, employment, and consumption, is less certain.

The mechanisms of this proposed association are not well understood, but poverty and mental ill health are hypothesized to operate in a negative cycle (Lund *et al*. 2010). The onset of mental illness may increase the risk of poverty (social drift), and conversely, the experience of poverty may increase the risk of mental ill health (social causation). Or it could be the case that the association between poverty and mental health is driven by third factors related to both poverty and mental illness, such as exposure to violence and poor physical health (Das *et al*. 2007). Another hypothesis is that poverty leads to stress and negative affect (social causation) and, in turn, stress and negative affect increase risk aversion which could make it harder for people to make the investments needed to escape poverty (Haushofer & Fehr, 2014).

One policy response to social drift (i.e. mental illness increases one's risk of poverty) is to increase access to mental health treatment, and the available evidence (although still quite limited) suggests that such interventions can improve economic outcomes (Lund *et al*. 2011). From this perspective, poor mental health is portrayed as a barrier to economic development (Miranda & Patel, 2005; Prince *et al*. 2007; Thornicroft & Patel, 2014).

While there is a strong ethical argument to be made for increasing access to mental health treatment (Prince *et al*. 2007), the fact remains that spending on mental health services in developing countries is currently less than US$0.25 per person per year and there exists a severe shortage in human resources (WHO, 2011), making the road to universal access long. For this reason, it would be beneficial if broad based poverty alleviation programs could have a positive impact on mental health and represent a pathway for indirect effects. In other words, evidence to support the social causation hypothesis that poverty leads to mental ill health would present an opening to invest in poverty alleviation programs as an indirect method of improving mental health outcomes.

The evidence base for this causal pathway is more limited, however, and the existing results are mixed. Lund *et al*. (2011) screened nearly 4000 abstracts related to this question and ultimately reviewed five articles published before 2010 that described the results of randomized controlled trials conducted in South Africa, Mexico, Ecuador, and Uganda. The economic interventions assessed included a loan program, cash transfer programs, and asset promotion program. All but one study, the randomized trial of a loan program in South Africa (Fernald *et al*. 2008), assessed only child outcomes.

In the one study that focused on adult outcomes, Fernald *et al*. (2008) randomized South Africans who had been rejected for loans to ‘second look’ evaluations with loan officers, and 53% of second look evaluations resulted in loans. Credit access had no impact on depressive symptoms overall but did increase perceived stress. A subgroup analysis suggested that credit access decreased depressive symptoms among men.

More recently, Haushofer & Shapiro (2013) randomized Kenyan villages and households to receive unconditional cash transfers of US$0, $400, or $1500 and found that the transfers had a positive impact on self-reported distress and depression among adults; recipients of the largest transfers also exhibited reduced levels of the stress hormone cortisol. Similarly, Ozer *et al*. (2011) compared Mexican women who participated in *Oportunidades*, a government-sponsored conditional cash transfer program, to a matched sample of women not exposed to the program and found that women in the treatment group had lower self-reported depression scores. The authors also presented evidence that this quasi-experimental effect was mediated by reductions in perceived stress and increases in perceived control.

There is a need for more rigorous evidence on whether and how poverty alleviation interventions can impact mental health symptoms, particularly in post-conflict settings where livelihoods have stalled and the prevalence of mental health disorders is often elevated (Tol *et al*. 2011). To contribute to this evidence base, we collaborated with AVSI, an Italian non-governmental agency working in Northern Uganda, to evaluate the impact of an economic assistance initiative on symptoms of depression via a cluster randomized trial. This program and evaluation began in 2009 as people were in the process of leaving crowded camps and returning home after a protracted displacement. From 1986 until 2005, the government and rebel groups, most notoriously the Lord's Resistance Army, had waged a war that resulted in mass internal displacement and left the majority of the population of Northern Uganda impoverished. Young women in particular suffered from the loss of economic and educational opportunities (Annan *et al*. 2011). The population was exposed to high levels of violence over two decades, and a representative study conducted near the end of the war found high levels of symptoms of traumatic stress and depression (Vinck *et al*. 2007). We hypothesized that a program of business skills training, cash grants of approximately US$150, and ongoing follow-up support would increase household income and, as a result, reduce symptoms of depression.

## Method

### Intervention

The Women's Income Generating Support (WINGS) program had three core components: (i) 5 days of business skills training designed for illiterate populations, (ii) an individual start-up grant of roughly US$150, and (iii) 3 to 5 visits over approximately 18 months by trained community workers who provided business advice and encouragement to use the grant for business development. The program was implemented and evaluated during a period of improved security and resettlement following the conflict. The details of the intervention are described elsewhere (Blattman *et al*., forthcoming).

### Participants

At the time of recruitment in 2009, a majority of the nearly 2 million people displaced by the conflict had left large internal displacement camps and resettled in smaller ‘transit’ camps closer to home or returned to villages of origin (IDMC, 2012). The agency invited 120 communities from a sampling frame consisting of 252 villages, transit sites, and displacement camps in Gulu and Kitgum districts to participate in WINGS. Communities were eligible for selection if the population was greater than 400 or if there were at least 80 households present. The number of communities chosen in each parish (an official subdivision of the subcounty) was decided based on the percentage of the district population present in those parishes. Because many displacement camps were in the process of closing down and encouraging residents to return to their villages, the agency restricted selection of displacement camps to those that were villages prior to becoming camps.

**Fig. 1.**
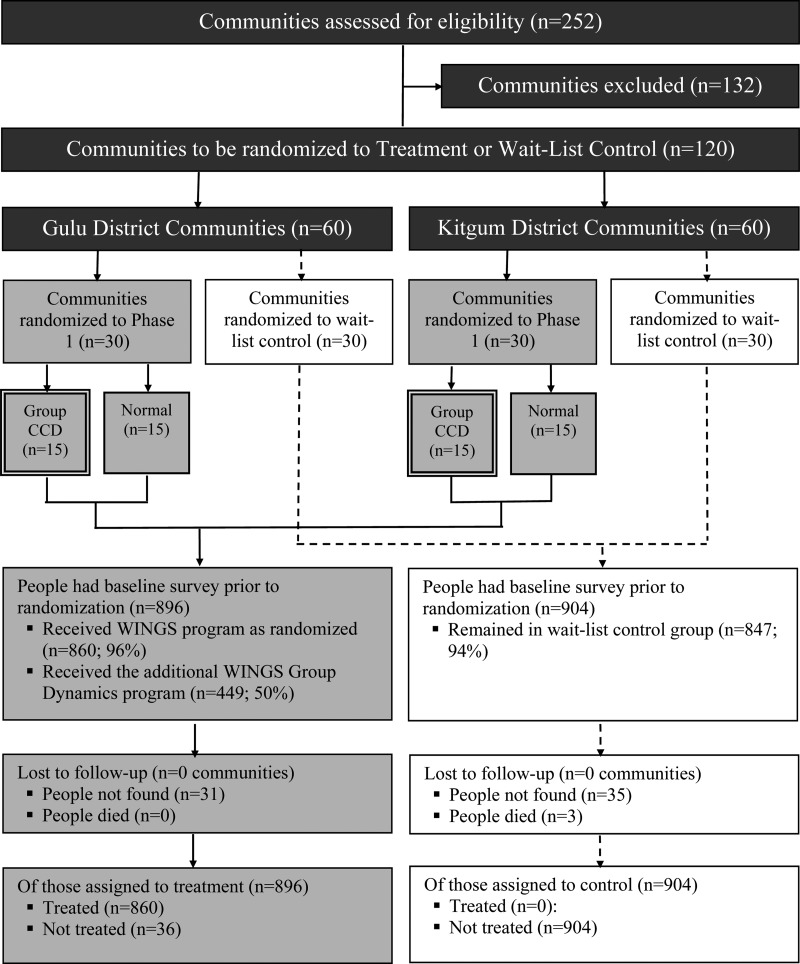
CONSORT-style flow diagram. ‘Group CCD’ refers to a cross-cutting design in which half of the treatment villages were assigned to also receive training and support to create business support groups. The results of this randomization are not reported here.

Once the sites were selected, the agency began a 2-month process of community sensitization and community-led beneficiary identification. Communities were asked to nominate approximately 20 of the poorest and most vulnerable members to participate in the program, predominantly young women (75%) between the ages of 14 and 30. A total of 2300 potential beneficiaries were identified across the project sites. Once identified, the agency conducted a preliminary assessment with 2280 individuals. The assessment protocol contained questions about participants’ household characteristics, physical health, well-being, social profile, and income generation capacity. The agency reviewed each case and selected 1800 individuals (approximately 900 per district) to be WINGS beneficiaries, with the goal of selecting 15 individuals in each community. The research team was not involved in beneficiary selection. See Fig. 1 for a CONSORT-style participant flow diagram.

### Measures

We assessed symptoms of depression using a modified version of the Acholi Psychosocial Assessment Instrument (APAI) depression subscale, a 35-item instrument developed in Northern Uganda (Bolton *et al*. 2007). The APAI is a one-dimensional measure of depression, but the scale items represent three local depression problems – *par, two tam*, and *kumu*. In the original validation study conducted in Northern Uganda, the APAI depression scale had a Cronbach's alpha of 0.92, test-retest reliability of 0.84, and evidence of concurrent validity with youth-caregiver dyad assessments of depression syndromes designed to distinguished cases of youth depression from non-cases.

We administered 34 of the original 35 APAI depression symptoms (excluding ‘loses concentration in school’ because it was not relevant to an out-of-school population), and respondents reported on a 4-point scale whether they experienced symptoms often (3), sometimes (2), rarely (1), or never (0). We assessed the structure of this subscale via confirmatory factor analysis (CFA) and found that a one-factor model with all of the items was not a good fit to our data. Therefore, we randomly split the baseline dataset into ‘training’ and ‘test’ halves and investigated alternative factor structures via exploratory factor analysis using the ‘training’ half of the dataset. We extracted three factors consisting of 19 items using principal axis factoring on the basis of a visual examination of a scree plot and the results of a parallel analysis and calculation of the optimal coordinate. Based on the results of a CFA using the ‘test’ half of the dataset, we constructed a one-dimensional depression score similar to the original APAI depression scale, only with a reduced set of items.

The total score on the reduced scale (APAI-R) had a possible range of 0–57, though we opted in the following analyses to report the total score as an average for easier interpretability, thus putting the APAI-R score on the original 0 to 3 metric corresponding to responses ranging from ‘never’ to ‘often’. Bolton *et al*. (2007) identified a threshold of 32 as ‘a reasonable lower bound for clinically significant presence of symptoms’ on the 35-item APAI (range 0–105). When averaged by the number of items, this cutoff translates to a score of 0.91 on the 0–3 scale.

We also collected data on several measures of economic security, including: self-reported household cash earnings in the last 4 weeks (profits), self-reported household savings, an indicator of business ownership, and an index of wealth based on participants’ reported dwelling characteristics, personal assets, livestock, and crops. We constructed a measure of endline business success based on this index of wealth, reported cash earnings, and non-durable consumption (i.e. goods purchased for frequent consumption, such as food, fuel, and clothing).

In addition to depression and economic security, we collected data on household characteristics, participant demographics, access to credit, physical health, risk aversion, support from neighbors and family, war experiences, community participation, and human capital. See Blattman *et al.* (forthcoming) for a complete description of secondary outcomes measures.

Survey items were translated into Luo by a faculty member at Makere University and was blind back-translated into English by an independent researcher in Gulu. The enumeration team assisted in reconciling any mistranslations in the Luo version of the survey and original English.

### Randomization

AVSI could only serve 900 individuals from 60 villages at once, so we planned for two program phases. Following a baseline survey with all 1800 enrolled beneficiaries in April 2009, we held a public lottery to randomly assign all 120 villages to receive the program in Phase 1 (immediate treatment) or the Phase 2 (wait-list control, delayed treatment). Randomization was stratified by district (60 villages per district), and villages were randomized to the immediate treatment group (60 villages with 896 participants) or the wait-list control group (60 villages with 904 participants) with an allocation ratio of 1:1. Participants, implementers, and data collectors were not blinded to treatment status.

### Procedures

Once the villages were randomized to Phase 1 (immediate treatment) or Phase 2 (wait-list control), the first phase of the program was implemented in the treatment villages. After Phase 1 concluded in late-2011, we conducted an endline survey with beneficiaries in all villages (treatment and control) approximately 16 months after receiving the initial training and grant, and prior to launching Phase 2 of the program in the wait-list control villages. All 1800 participants completed the baseline survey, and there was very little missing data (<0.5%). To avoid losing the observation, we imputed missing baseline data with the sample median. Attrition at the Phase 1 endline was 3.7% and uncorrelated with assignment to treatment.

All study procedures were reviewed and approved by the Uganda National Council for Science and Technology and Institutional Review Boards at Yale University and Columbia University. Participants gave informed consent to enroll in the study. This study was not pre-registered. When the study began in 2009, very few studies of economic assistance programs were pre-registered in medical registries like ClinicalTrials.gov. Other registries like RIDIE (3ie), the American Economic Association's RCT registry, and the Experiments in Governance and Politics Network (EGAP) registry did not exist at the time.

### Statistical analysis

#### Correlates of depression

We examined predictors of depression by regressing baseline depression scores on participant demographics and household characteristics, indicators of economic security, physical health, risk aversion, support from neighbors and family, war experiences, community participation, and human capital.

#### Estimating treatment effects

With 120 clusters, 15 participants per cluster, *α* = 0.05, (1 − *β*) = 0.80, *ρ* = 0.12, and *R*_12_ = 0, this study was powered to detect a minimum effect size of 0.2, which is roughly equivalent to a difference score of 0.11 on the APAI-R (range 0–3). The intent-to-treat analysis included 860 treatment participants (of 896) and 866 control participants (of 904). Standard errors were clustered at the village-level and the ordinary least squares regression included dummy variables for district strata. We tested for heterogeneity in the treatment effects on symptoms of depression and business success according to pre-treatment levels of depression (interaction of indicator for assignment to treatment and pre-treatment levels of depression).

## Results

Participant characteristics are displayed in Table 1. At baseline, the mean age of the sample was 27.3 (s.d. = 7.2), and females made up 86.2% of all participants (s.d. = 34.5%). On average, participants completed 2.8 years of schooling (s.d. = 2.8), and roughly half reported that they were currently married (47.9%; s.d. = 0.5). There was a small but statistically significant difference between the treatment and wait-list control group in baseline scores on the measure of symptoms of depression: the unadjusted treatment mean is 0.85 and the wait-list control mean is 0.75, *p* < 0.05. Due to this difference, we controlled for baseline scores in the impact analysis. Both group means approach the APAI cutoff for clinical significance of 0.91.

**Table 1.** Baseline participant characteristics

	Baseline (*n* = 120 villages)	
Variables	Treatment (s.d.)	Control (s.d.)
Participant characteristics		
Age	27.02 (7.20)	27.63 (7.29)
Female (0–1)	0.86 (0.35)	0.86 (0.35)
Years of schooling	2.77 (2.83)	2.91 (2.86)
Married/cohabiting (0–1)	0.46 (0.50)	0.50 (0.50)

Treatment compliance was high and survey attrition was low [see Blattman *et al.* (forthcoming) for details]. We found 96.3% of the sample at endline, and attrition was generally not significantly correlated with treatment or baseline covariates. Roughly 96% of people assigned to treatment (and no one assigned to wait-list control) received the training or grant in Phase 1. No clusters were lost.

### Correlates of depression

As shown in Table 2, wealth is negatively associated with APAI-R scores at baseline, and this relationship is statistically significant. Other significant protective factors include access to credit and household support. Significant risk factors for symptoms of depression include being female, having a larger household size, being food insecure, and having more war experiences. Counter-intuitively, good physical health is also positively associated with APAI-R scores.

**Table 2.** Multiple regression of baseline APAI-R score on household and respondent characteristics

Variable	*B* (s.e.)	*t*
(Intercept)	0.26 (0.078)	3.3***
Female (0/1)	0.12 (0.035)	3.3***
Age	0.00 (0.002)	1.3
Non-Acholi (0/1)	0.09 (0.061)	1.4
One/both parents died before age 15 (0/1)	0.06 (0.037)	1.8
Sole earner in household (0/1)	0.04 (0.023)	1.7
Household size	0.03 (0.006)	5.0***
Average weekly work hours	0.00 (0.001)	−2.8**
Wealth index (*z*)	−0.17 (0.029)	−5.8***
Credit index (*z*)	−0.03 (0.012)	−2.1*
Health index (*z*)	0.13 (0.014)	9.3***
Food insecure (0/1)	0.08 (0.026)	3.0**
Risk aversion (*z*)	0.01 (0.012)	0.8
Household support (*z*)	−0.08 (0.013)	−6.4***
Neighbor support (*z*)	−0.01 (0.015)	−0.7
War experiences (*z*)	0.07 (0.014)	5.3***
Community participation (*z*)	0.00 (0.015)	0.0
Human capital (*z*)	−0.01 (0.015)	−0.4

Note: This table reports the results of an ordinary least squares regression of baseline APAI-R scores on respondent and household characteristics. s.e. clustered at village level. APAI-R scores have a possible range of 0 to 3, where 3 represents more severe self-reported depressive symptoms. Respondent age is mean centered. *F*(17,1726) = 25.6, *p* < 0.001, Adjusted *R*^2^ = 0.20.

*p < 0.05, **p < 0.01, ***p < 0.001.

### Treatment effects

Sixteen months after the baseline survey, monthly cash earnings doubled from UGX 15 529 to 31 842 (purchasing power parity US$1.00 to UGX 800), non-household and non-farm businesses doubled, and cash savings roughly quadrupled (see Table 3). While these effects are small in absolute terms, they are large and meaningful relative to where the participants start, moving the study participants from the bottom of the local income distribution to the middle. See (omitted for review) for a full discussion of the economic impacts.

**Table 3.** Primary outcomes

	Baseline (*n* = 120 villages)	Endline (*n* = 120 villages)		
Variables	Mean (s.d.)	Mean (s.d.)	ATE (s.e.)	Obs
APAI-R (0–3)				
Treatment group	0.85 (0.56)	0.60 (0.50)	−0.03 (0.03)	1732
Control group	0.75 (0.53)	0.59 (0.51)		
APAI-R above 0.91 cutoff (0/1)				
Treatment group	0.40 (0.49)	0.24 (0.42)	−0.02 (0.24)	1732
Control group	0.32 (0.47)	0.23 (0.42)		
APAI-R response (>50% reduction)				
Treatment group		0.38 (0.49)	0.03 (0.3)	1732
Control group		0.34 (0.47)		
Engaged in petty trading (0/1)				
Treatment group		0.80 (0.40)	0.40 (0.03***)	1729
Control group		0.39 (0.49)		
Cash earnings last 4 weeks (UGX)				
Treatment group		31 842 (73 155)	10 386 (3454**)	1734
Control group		15 529 (36 800)		
Wealth index (*z*-score)				
Treatment group	−0.67 (0.45)	0.37 (0.87)	0.39 (0.07***)	1734
Control group	−0.61 (0.47)	0.07 (0.87)		
Business success (*z*-score)				
Treatment group		0.23 (1.05)	0.46 (0.07***)	1734
Control group		−0.22 (0.89)		

Note: The average treatment effect (ATE) is the coefficient on the assignment to treatment in a regression of the primary outcome on treatment assignment (intention to treat) and a set of baseline controls and district dummy (not shown).

s.e. are clustered at the village-level.

*p < 0.05, **p < 0.01, ***p < 0.001.

We observed decreases in depression severity in both groups over time. At endline, we observed that the treatment group mean decreased by 29%, from 0.85 to 0.60. Similarly, the control group mean decreased by 21%, from 0.75 to 0.59. As a reference, Bolton *et al*. (2007) define ‘recovery’ (response) as ‘a reduction of 50% or more of an individual's baseline symptom severity score’.

The average treatment effect on symptoms of depression, however, is small and not statistically significant (see Table 3). In a regression of average scale scores on treatment assignment and a set of baseline controls, including baseline symptoms of depression, the coefficient on the assignment to treatment is −0.03. Similarly, there is no evidence of impact on: (i) the proportion of participants with APAI-R scores above the cutoff for clinical clinically significant symptom levels or (ii) the proportion of participants exhibiting a 50% reduction in APAI-R scores from baseline (response). This pattern holds even among the subset of participants with APAI-R scores above the cutoff at baseline (see Table 4). Additionally, there is no evidence that pre-treatment symptoms of depression moderate business success or the program impact on depressive symptoms.

**Table 4.** Treatment heterogeneity

Pre-treatment variables	Business success		APAI-R		APAI-R Response (>50% reduction from baseline)	
Assigned to treatment (0/1)	0.47 (0.07)***	0.45 (0.08)***	−0.01 (0.03)	0.00 (0.03)	0.05 (0.03)	0.01 (0.04)
APAI-R > 0.91 cutoff (0/1)		−0.01 (0.06)		0.21 (0.03)***		0.20 (0.04)***
(APAI-R > 0.91 cutoff) × (assigned to treatment)		0.05 (0.09)		−0.04 (0.05)		0.05 (0.05)
Constant	−0.53 (0.36)	−0.51 (0.37)	0.01 (0.19)	−0.01 (0.18)	0.85 (0.18)***	0.84 (0.18)***
Observations	1734	1734	1732	1732	1734	1734
*R*^2^	0.25	0.25	0.22	0.25	0.08	0.13
Control group mean	−0.22	−0.22	0.59	0.59	0.34	0.34

Robust s.e. in brackets, clustered by village. Baseline controls and district dummy omitted in table.

*p < 0.05, **p < 0.01, ***p < 0.001.

## Discussion

This study reports the findings of a cluster-randomized trial of an economic assistance program in post-conflict Northern Uganda on symptoms of depression. Despite finding large increases in business activity and reductions in poverty among the treatment group, we found no measurable endline difference between the groups in reported symptoms of depression.

Our results make several important contributions to the literature on poverty and mental health. First, we demonstrate an association between baseline wealth and symptoms of depression, thus adding to the large body of work in low-income countries that suggests that poverty and mental health are in fact related (Patel & Kleinman, 2003; Lund *et al*. 2010). This association is present even among the most poorest and most marginalized. Second, we find that the program led to a large economic impact that was not moderated by preexisting symptoms of depression, thus supporting the view that mental illness should not be presumed to be a barrier to helping the most vulnerable to secure small amounts of capital and training to support income-generating activities. Third, we add to the evidence base about the indirect effect of poverty alleviation on mental ill health. Like Fernald *et al*.'s (2008) study of ‘second look’ loan evaluations in South Africa, we find no effects of entrepreneurship assistance on depressive symptoms. In the current study, we find this null result despite clear evidence that the program led to large increases in income, consumption, and wealth. These null findings stand in contrast, however, to two studies of conditional and unconditional cash transfers in Kenya (Haushofer & Shapiro, 2013) and Mexico (Ozer *et al*. 2011) that reported effects on depression.

Since the program we studied led to large increases in household's economic well-being, and given that treatment compliance was high and attrition was low and largely non-systematic, this null finding is informative. It is possible that the gains women derived from increased economic security were offset by stressors associated with planning, launching, and maintaining a new business. This interpretation would fit with Fernald *et al*.'s (2008) finding that second chance loans in South Africa were associated with increased perceived stress.

It could also be the case that the economic improvement reported by participants might not have been enough to alleviate this related stress. This would fit with Haushofer & Shapiro's (2013) finding that small and large unconditional cash transfers reduced depression, but only large transfers reduced levels of cortisol. It makes intuitive sense that unconditional cash transfers that provide immediate benefits without the worries associated with loan repayments or the need to earn profits could potentially have a stronger impact on mental wellbeing than conditional transfers and high-interest loans.

Finally, we note that this study took place during a period of rapid social and economic change. Both the treatment and the control group reported substantial reductions in the severity of depression symptoms, which lends support to the idea that income may not drive the relationship between poverty and mental health (Patel & Kleinman, 2003; Lund *et al*. 2010). The increased sense of security, hope for the future, and new opportunities that came with a recovering economy might have overshadowed any effect of increasing household income.

### Limitations

This study has several limitations. First, it is unknown to what extent these results would generalize to other post-conflict settings. Second, the study sample was not drawn from a clinical population, so it is unknown whether the same pattern of results would be found if depressed patients were explicitly targeted for assistance. Third, our decision to use a locally derived measure of depression symptoms limits comparisons to other studies.

## Conclusions

Even among the poorest segments of society, deprivation and depression covary. The mechanisms that might explain this link between poverty and mental health, however, remain uncertain (Burns, 2015). Some evidence suggests that unconditional cash transfers reduce depression in low-income settings, while other evidence suggests that loans and entrepreneurship support might not be as effective. Additional evidence is greatly needed to guide donors and policy makers in considering the role of poverty alleviation programs in addressing the treatment gap for CMD in low-income countries, particularly in post-conflict settings.
